# Room temperature XFEL crystallography reveals asymmetry in the vicinity of the two phylloquinones in photosystem I

**DOI:** 10.1038/s41598-021-00236-3

**Published:** 2021-11-08

**Authors:** Stephen M. Keable, Adrian Kölsch, Philipp S. Simon, Medhanjali Dasgupta, Ruchira Chatterjee, Senthil Kumar Subramanian, Rana Hussein, Mohamed Ibrahim, In-Sik Kim, Isabel Bogacz, Hiroki Makita, Cindy C. Pham, Franklin D. Fuller, Sheraz Gul, Daniel Paley, Louise Lassalle, Kyle D. Sutherlin, Asmit Bhowmick, Nigel W. Moriarty, Iris D. Young, Johannes P. Blaschke, Casper de Lichtenberg, Petko Chernev, Mun Hon Cheah, Sehan Park, Gisu Park, Jangwoo Kim, Sang Jae Lee, Jaehyun Park, Kensuke Tono, Shigeki Owada, Mark S. Hunter, Alexander Batyuk, Roland Oggenfuss, Mathias Sander, Serhane Zerdane, Dmitry Ozerov, Karol Nass, Henrik Lemke, Roman Mankowsky, Aaron S. Brewster, Johannes Messinger, Nicholas K. Sauter, Vittal K. Yachandra, Junko Yano, Athina Zouni, Jan Kern

**Affiliations:** 1 Molecular Biophysics and Integrated Bioimaging Division, Lawrence Berkeley National Laboratory, Berkeley, CA 94720 USA; 2 Institut für Biologie, Humboldt-Universität Zu Berlin, 10115 Berlin, Germany; 3 LCLS, SLAC National Accelerator Laboratory, Menlo Park, CA 94025 USA; 4 National Energy Research Scientific Computing Center, Lawrence Berkeley National Laboratory, Berkeley, CA 94720 USA; 5 Department of Chemistry - Ångström, Molecular Biomimetics, Uppsala University, 75237 Uppsala, Sweden; 6 Department of Chemistry, Umeå University, Linnaeus väg 6 (KBC huset), 90187 Umeå, Sweden; 7 Pohang Accelerator Laboratory, POSTECH, Pohang, 37673 Korea; 8 Japan Synchrotron Radiation Research Institute, 1-1-1 Kouto, Sayo-cho, Sayo-gun, Hyogo, 679-5198 Japan; 9 RIKEN SPring-8 Center, 1-1-1 Kouto, Sayo-cho, Sayo-gun, Hyogo, 679-5148 Japan; 10 Paul Scherrer Institut, 5232 Villigen, Switzerland; 11 Present Address: Department of Bioengineering and Therapeutic Sciences, University of California, San Francisco, CA 94158 USA

**Keywords:** Biochemistry, Structural biology, Bioenergetics, Photosynthesis

## Abstract

Photosystem I (PS I) has a symmetric structure with two highly similar branches of pigments at the center that are involved in electron transfer, but shows very different efficiency along the two branches. We have determined the structure of cyanobacterial PS I at room temperature (RT) using femtosecond X-ray pulses from an X-ray free electron laser (XFEL) that shows a clear expansion of the entire protein complex in the direction of the membrane plane, when compared to previous cryogenic structures. This trend was observed by complementary datasets taken at multiple XFEL beamlines. In the RT structure of PS I, we also observe conformational differences between the two branches in the reaction center around the secondary electron acceptors A_1A_ and A_1B_. The π-stacked Phe residues are rotated with a more parallel orientation in the A-branch and an almost perpendicular confirmation in the B-branch, and the symmetry breaking PsaB-Trp673 is tilted and further away from A_1A_. These changes increase the asymmetry between the branches and may provide insights into the preferential directionality of electron transfer.

## Introduction

**Figure 1:**
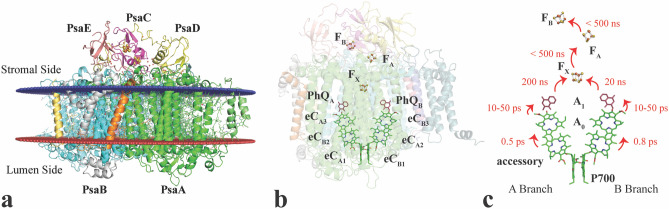
Overview of PS I. (**a**) Membrane plane view of one monomer of PS I with multiple subunits represented in different colors. The two main subunits PsaA and PsaB and the three membrane extrinsic subunits PsaD, PsaC, and PsaE are labeled. Many of the trans-membrane helices are visible between the membrane planes (red and blue planes). Membrane orientation generated via the PPM server (https://opm.phar.umich.edu/ppm_server). (**b**) View of the reaction center part of PS I. The cofactors involved in electron transfer are arranged in two pseudo-symmetrical branches comprised of Chl eC_A1_, eC_B2_, eC_A3_ and PhQ_A_ on one side and Chl eC_B1_, eC_A2_, eC_B3_ and PhQ_B_ on the other side. Both branches connect at the [4Fe4S] cluster F_X_ at the stromal side, with F_A_ and F_B_, the two final [4Fe4S] clusters, breaking the symmetry. (**c**) Electron transfer times along the A and B-branch of the reaction center of PS I. Cofactors are labeled according to their function, with P700 and the accessory Chls forming the primary donor, A_0_ being the first and A_1_ the secondary electron acceptor in the electron transfer chain on each branch. Figure generated with Pymol v. 2.4.0a0. https://pymol.org.

Photosystem I (PS I) is a large multisubunit pigment-protein complex involved in the light-driven electron transport across the thylakoid membrane that is present in all organisms that perform oxygenic photosynthesis. It consists of two large membrane intrinsic subunits (PsaA and PsaB) with 11 transmembrane helices each, which are surrounded by several smaller membrane intrinsic subunits, namely PsaF, PsaI, PsaJ, PsaK, PsaL, PsaM and PsaX in *Thermosynechococcus* *(T.)* *elongatus*. At the stromal side of the thylakoid membrane, PS I displays three membrane extrinsic subunits (PsaC, PsaD, PsaE) (Fig. 1). For cyanobacterial PS I, the complex usually forms a trimer (Fig. S1) with a total molecular weight of ~ 1 MDa^1^. The structure for a monomer of cyanobacterial PS I was found to be very similar to the structure of PS I found in higher plants^2–4^ and algae^5, 6^ with the important difference that in plant and algae, PS I occurs exclusively in a monomeric form and is surrounded by an array of light-harvesting complexes. Over the last 20 years, several PS I structures have been reported from different organisms at cryogenic temperatures. The first structure was from the cyanobacterium *T.* *elongatus*, initially at 6 Å resolution^7^ with subsequent improvements to 4 Å^8, 9^, and finally leading to a structure at 2.5 Å resolution^1^.

The internal antenna system of PS I comprises ~ 90 chlorophyll a (Chl) and 22 β-carotenoids (Car). The central two subunits PsaA and PsaB harbor the cofactors of the transmembrane electron transfer chain in a symmetrical fashion, along two pseudo-symmetrical branches, similar to the arrangement found in the purple bacterial reaction center (PBRC)^10^. The A branch of PS I contains chlorophylls eC_A1_, eC_B2_, eC_A3_ and phylloquinone PhQ_A_, and the B branch contains chlorophylls eC_B1_, eC_A2_, eC_B3_ and phylloquinone PhQ_B_ (Fig. 1b). Upon light absorption, the excitation energy is transferred from the antenna via the connecting chlorophylls towards P700, where charge separation is initiated within ~ 1 ps. The identity of the first donor Chl is still debated^11^, with P700^12^ or the accessory Chl^13–15^ as candidates (eC_B2_ or eC_A2_, Fig. 1c). Within 10–50 ps the electron is transferred from the acceptor chlorophyll A_0_ to A_1_, a phylloquinone (PhQ) resulting in the formation of the semi phylloquinone PhQ^—^ P700^+^ pair^12–15^. Next, the electron is transferred with two distinct phases of 20 and 200 ns to the central [4Fe4S] cluster termed F_X,_ which is located at the interface between the A and B subunits and is coordinated by two Cys residues from each of the two subunits. Finally, the electron is transferred to the two [4Fe4S] clusters in subunit PsaC, termed F_A_ and F_B_ (Fig. 1c, see also e.g. Kurashov et al*.*^16^). These can, in turn, donate the electron to the external electron carrier, a ferredoxin. EPR studies on co-crystals of PS I with ferredoxin indicated intermittent binding to the surface of PsaC that is exposed towards the stroma^17^, and was confirmed in recent structures of ferredoxin bound to PS I^18, 19^.

The structure of the reaction center found in PS I with the arrangement of the central cofactors in two pseudo-symmetrical branches, both starting at P700 and ending at F_X_, led to the question of how this electron transfer proceeds (it should be noted that P700 is not a symmetric Chl dimer but one Chl is a Chl a′, an epimer of chlorophyll a). Electron transfer could either proceed along one branch solely, along both branches equally, or along both branches with different ratios. Previous crystal structures of PS I showed that the symmetry of the two branches is broken by the link between the last acceptor F_X,_ and the preceding phylloquinone in the electron transfer chain; with Trp673 present in the B branch, and Glycine in the A branch^1^. Also, a difference in the nature of lipids located close to PhQ_A_ and PhQ_B_ was observed, with a negatively charged phospholipid located 10 Å away from PhQ_B_, and an uncharged galactolipid located ~ 11 Å away from PhQ_A_^20^.

A combination of spectroscopic and mutagenesis studies revealed that both branches are active and that the 200 ns phase is associated with the A branch^21^. Femtosecond transient absorption spectroscopy further showed that the primary charge separation step can occur on both branches and that they compete with each other^14^. There are different results regarding the partitioning ratio and ET rates along the two branches^22^; depending on the species and experimental conditions, branching ratio of 90:10–44:56% (for A and B branches, respectively) have been reported^22–25^. Kinetic and energetic differences between the two branches have been the topic of numerous studies. However, details of the mechanisms and clear experimental evidence for the various models are presently a subject of contention. Crystallographic data measured at physiological conditions may assist in answering some of the outstanding unknowns regarding electron transfer directionality in this system.

The regulation and fine-tuning of cofactor properties (especially the redox potential) by the interaction with the protein environment are key to direct the flow of electrons in photosynthetic protein assemblies like PS I. Time-resolved crystallography is an ideal tool to study structural changes that underlie and stabilize light-induced charge separation, and can potentially help to better understand the role of the protein environment for determining cofactor properties. With the advent of X-ray free-electron lasers (XFELs) over the last 10 years, serial femtosecond crystallography (SFX) has developed into a powerful tool to obtain structures at room temperature (RT), without accompanying X-ray induced structural changes (“diffraction before destruction”)^26–28^. The method pioneered by Fromme, Spence, Chapman, Neutze and coworkers using micro crystals of PS I, determined the first RT structure of PS I at ~ 8 Å resolution by recording thousands of individual diffraction patterns, each obtained from a new micro crystal delivered to the X-ray interaction point by a gas-focused liquid jet^20^. Recently, an SFX crystal structure of PS I was obtained by data collected at the European XFEL^29^. The 2.9 Å resolution structure demonstrated the proof of concept that data from complex, membrane-bound proteins could be measured using MHz X-ray repetition rates; however, owing to resolution limits, much structural information cannot be interpreted from these maps. Interestingly the authors of this study reported a space group P2_1_ for their PSI microcrystals as opposed to the P6_3_ space group previously reported for the cryogenic data^1^.

It should be emphasized that changes in temperature can cause important structural differences that could be relevant for enzymatic function, as evidenced in studies by Fraser and van den Bedem et al., comparing traditional cryogenic data collection at 100 K with results from RT measurements^30^, see also Ref.^31^ for a recent example of differences observed between XFEL RT and synchrotron cryogenic structures. These studies were mostly focused on soluble proteins, whereas only limited information is available for temperature-dependent structural changes in membrane proteins^30, 32^. In the recent work by Young et al., it was found that, for the membrane protein complex PS II, a uniform expansion of the entire complex along the membrane plane by about 0.5 Å occurs when comparing the room temperature structure to cryogenic structures^32^.

In order to allow efficient time-resolved X-ray diffraction studies at XFELs, we recently developed a Drop-on-Tape (DOT) setup^33^. This DOT setup allows optical pump X-ray probe experiments with delay times ranging from femtoseconds out to seconds, at room temperature, with very high sample hit rates leading to reduced measurement time and sample consumption.

Here, we studied the details of the cofactor–protein interaction in PS I at near-physiological conditions to gain insights into factors that are responsible for fine-tuning the electron transfer pathway in PS I. We utilized microcrystals of PS I from the thermophilic cyanobacterium *T. elongatus* with our DOT approach and XFEL generated fs X-ray pulses. Clear differences around the PhQ cofactors in both branches A and B, compared to the previously reported cryogenic structures were observed, possibly contributing to the differences in electron transfer rates along the two branches.

## Results

### Overall structure

In order to obtain an undamaged room temperature structure in the dark state of PS I, we utilized the fs X-ray pulses of the LCLS^34^ and the SwissFEL^35^. We used the DOT setup^33^ installed at the MFX instrument of LCLS^36^ or at the Bernina instrument at SwissFEL^35^ to measure X-ray diffraction data from microcrystals (15–25 µm) of trimeric PS I from *T. elongatus* (Methods, Fig. S2). The resulting data set was processed with the cctbx.xfel software suite^37^ (see Methods). Bragg spots were observed up to 2.3 Å resolution, with individual data sets evaluated for initial quality to determine merging resolution (Table S1). To improve quality and statistics of the overall data set, data was merged from both instruments to yield a data set to be sufficient for refinement and structure determination up to a resolution of 2.75 Å. Details about the data statistics and processing are given in Tables 1 and 2.

**Table 1:** Data processing and merging statistics PDB ID 7M75 7M78 7M76.

Beamline	LCLS/MFX + SwissFEL/Bernina	SACLA/BL2	PAL XFEL/NCI
Resolution range refined (Å)	56.71–2.75	31.63–3.00	25.23–3.00
Resolution range upper bin (Å)	(2.79–2.75)	(3.05–3.00)	(3.05–3.00)
Wavelength (Å)	1.306	1.181	1.277
Space group	P6_3_	P6_3_	P6_3_
Unit cell parameters (Å)	a = 285.4 ± 0.3 b = 285.4 ± 0.3 c = 166.5 ± 0.3	a = 284.9 ± 1.4 b = 284.9 ± 1.4 c = 166.2 ± 1.4	a = 284.3 ± 0.3 b = 284.3 ± 0.3 c = 165.8 ± 0.4
Lattices merged	143,459	22,560	5901
Unique reflections	199,647	152,883	147,785
(upper bin)	(9949)	(7619)	(7378)
Completeness	99.98%	99.9%	100%
(upper bin)	(99.88%)	(100%)	(100%)
CC_1/2_	99.8%	89.3%	90.4%
(upper bin)	(13.5%)	(9.8%)	(3.1%)
Multiplicity	506.39	282.5	32.0
(upper bin)	(9.84)	(62.31)	(12.71)
I/σ_Br19_(I)^##^	3.6	3.2	1.3
(upper bin)	(0.2)	(0.9)	(0.3)

^##^: as defined in [Brewster 2019]^79^.

In light of the recent report of an XFEL-based PS I structure from crystals in the space group P2_1_^29^, instead of the P6_3_ reported for the earlier structures^1^, we decided to thoroughly investigate the unit cell of our present PS I crystals. We obtained publicly archived raw data from the Coherent X-ray Imaging Database (CXIDB deposit 111) for the monoclinic structure reported by Gisriel et al.^29^. Ten runs (167–176) were reanalyzed as follows: The archived and our working data sets were compared by indexing runs starting with the lattice parameters of the P2_1_ structure (a = 279.1, b = 164.6, c = 284.1, beta = 119.25). Unit cells were refined on a per-crystal basis and histograms of the refined lattice parameters were constructed. Consistent with the previous report, the combined histograms for the *a* and *c* axes for the CXIDB 111 data form a bimodal distribution with maxima at 281.0 and 286.2 Å. In contrast, the corresponding histogram for our present data is unimodal with a maximum at 285.0 Å (Fig. S3). The refined *beta* angles are centered on 119.3°. for the CXIDB 111 data and on 119.9°. for the present data. Thus, our sample matches the hexagonal isoform in P6_3_ with a symmetric PS I trimer and not the monoclinic isoform in P2_1_. A single particle cryo-EM study^38^ recently demonstrated the threefold molecular symmetry of our PS I, which is a necessary (but not sufficient) condition for P6_3_ crystallographic symmetry. We conclude that crystallization may occur in a hexagonal isoform with molecular threefold symmetry (Jordan 2001^1^ and present work) or in a monoclinic isoform with molecular pseudo-threefold symmetry (Gisriel 2019)^29^.

**Figure 2:**
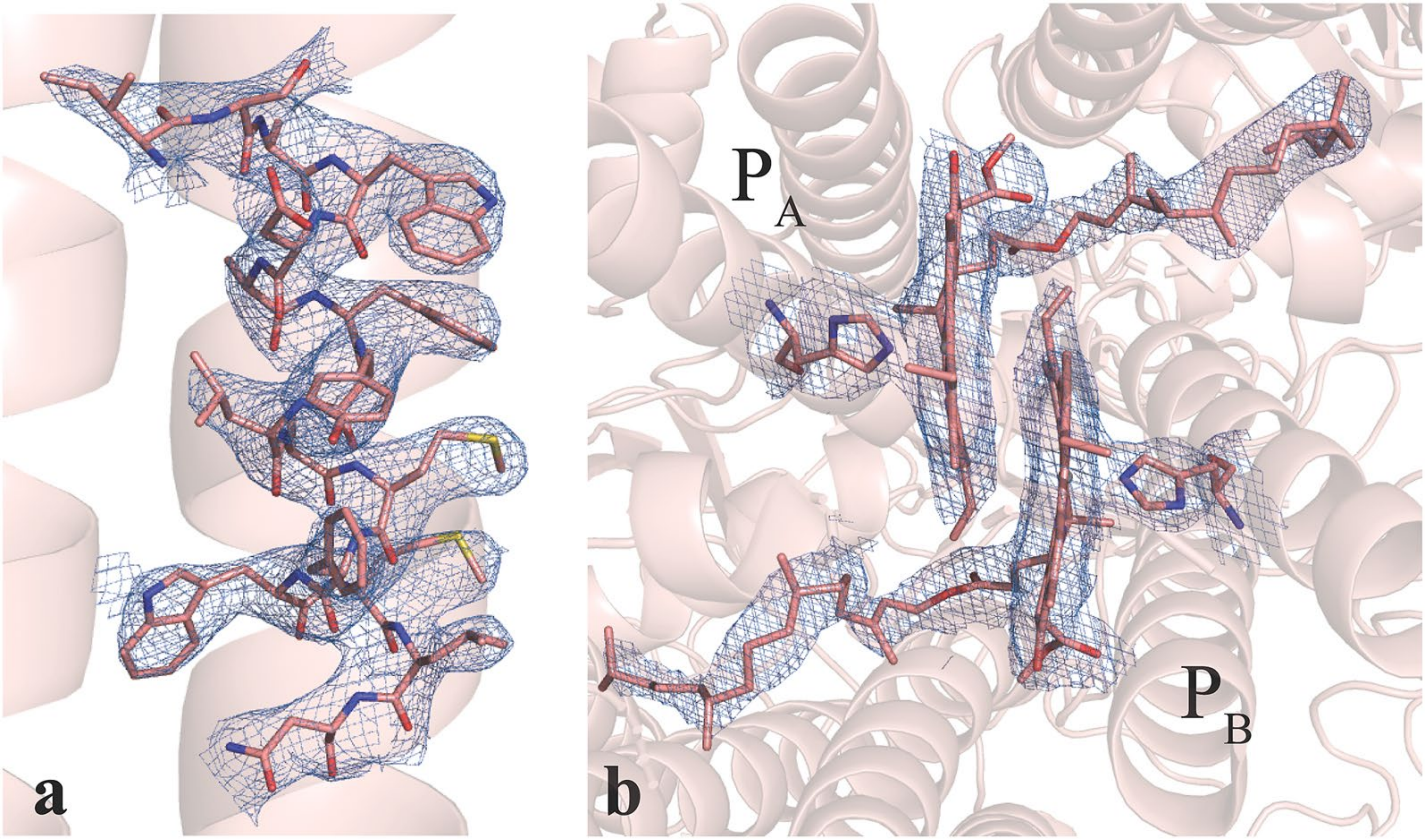
Electron density of selected regions of the PS I protein complex derived from the 2.75 Å resolution room temperature diffraction data. (**a**) A portion of a transmembrane alpha-helix (PsaB576–PsaB591) with side chains modeled into 2F_obs _− F_calc_ electron density contoured to 1.5 $\sigma$. Side chains are clearly visible in this region of the map. (**b**) Electron density in the region of the special pair P700 formed by a Chl a (labeled P_B_) and a Chl a’ (labeled P_A_). Mg^2+^ coordinating His residues and the Chls are shown as sticks, the 2F_obs _− F_calc_ electron density is shown as blue mesh contoured at 1.5 $\sigma$. Figure generated with Pymol v. 2.4.0a0, https://pymol.org.

Examples for assessing the quality of our data can be seen in Fig. 2, where the electron density for some selected regions of the protein is shown. The quality is as expected for a data set at 2.75 Å resolution and allows modeling of amino acid side chains and cofactor details. The quality of the electron density map was also of sufficient quality to model 4 additional lipids (e.g. Fig. S5) compared to the cryogenic structure. We modeled all 96 chlorophylls present in the cryogenic structure (1JB0) but we note that the chlorophyll in PsaM was modelled in a slightly different position compared to 1JB0 and has weak density allowing for alternative ligands to be modeled into this site.

**Figure 3:**
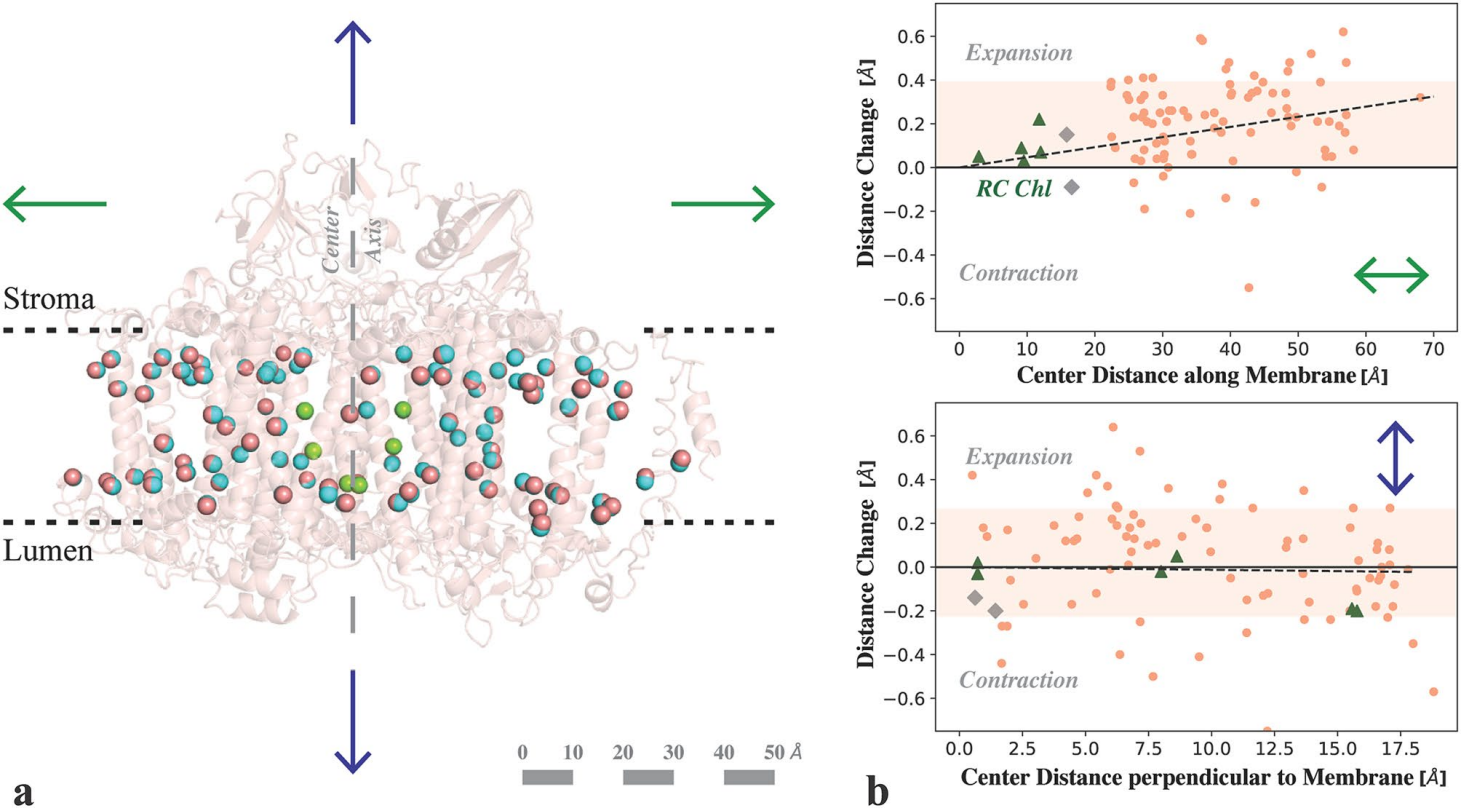
Comparison of Chl positions in PS I between cryogenic and RT structure. (**a**) A representation of the central Mg^2+^ (salmon spheres) of all Chls in one monomer of the PS I RT structure (with backbone shown schematically in salmon) overlaid with the Mg^2+^ positions from the cryogenic structure (blue). View is along the membrane plane with the stromal side on top and lumenal side on bottom. The center axis, passing through F_X_ and P700 is indicated by a long-dashed line. (**b**) Distance change (RT minus cryo) of the chlorophylls to the center between P700 and F_X_ either along the membrane (*top*) or perpendicular to it (*bottom*). Positive values represent expansion, negative contraction at room temperature. The 1-σ region around the average is highlighted. The Chls involved in charge separation are marked as green triangles, the closest two Chls of the antenna as gray diamonds. The room temperature structure shows an average expansion by about 0.2 Å along the membrane plane. The distance change perpendicular to the membrane is distributed symmetrically around the zero line. Figure generated with Pymol v. 2.4.0a0, https://pymol.org, Python v. 3.7.6, Matplotlib 3.2.1, https://matplotlib.org.

At a first glance the overall structure is very similar to the reported cryogenic structure of PSI reported by Jordan et al.^1^ (RMSD = 0.488 Å for C-α atoms). Yet upon closer inspection clear differences were observed. A small but reproducible expansion along the membrane plane of the overall structure is visible in the RT structure compared to the cryogenic structure, along with an expanded unit cell (a = 285.3, b = 285.3, c = 166.5). The radius of gyration of the monomer increases from 40.8 Å in the cryo-structure to 41.3 Å in the XFEL structure, and that of the trimer from 68.2 Å (cryo) to 68.7 Å (XFEL) (Fig. S1). The changes in the distance from the chlorophylls to the center between P700 and F_X_ are illustrated in Fig. 3. The average expansion at RT is 0.2 Å along the membrane plane with a gradual increase of about 0.05 Å per 10 Å (Fig. 3b top, dotted line). In contrast, distances perpendicular to the membrane show no average change (Fig. 3b, bottom). The expansion of the protein affects the distances between the antenna chlorophylls, and between the antenna and the core. Interestingly, no clear effect on the proposed connecting Chl (shown in grey in Fig. 3b) is visible. Cofactors involved in the electron transport, which is directed perpendicular to the membrane, are only marginally affected (eC_A/B1-3_ highlighted in green). Two separate RT data sets of PS I were collected the SACLA XFEL facility in Japan and in the presence of ascorbate at the PAL XFEL facility in Korea, and structural models were refined into these data sets to 3.0 Å resolution (Tables 1 and 2). In these independent experiments the PS I complex showed a similar expansion along the membrane plane when compared to the cryogenic structure (Fig. S4).

**Table 2:** Refinement statistics PDB ID 7M75 7M78 7M76.

Beamline	LCLS/MFX + SwissFEL/Bernina	SACLA/BL2	PAL XFEL/NCI
Wilson B-factor	68.4	41.1	74.5
R-factor	26.4%	33.4%	26.8%
R-free	27.9%	34.9%	27.8%
Number of atoms	48,898	48,799	48,799
Number non-hydrogen atoms	24,512	24,413	24,413
Ligands	14,127	14,127	14,127
Waters	119	20	20
Protein residues	2244	2244	2244
RMS (bonds)	0.006	0.003	0.006
RMS (angles)	0.991	0.66	1.08
Ramachandran favored	91.61%	93.5%	90.7
Ramachandran outliers	0.09%	0.05%	0.09%
Clashscore	15.5	7.5	14.8
Average B-factor	87.3	51.7	77.2

The positions of the uncharged glycolipid and negatively charged phospholipid located near the A branch and B branch respectively, closely match what was observed in previous cryogenic structures. The asymmetry these lipids introduce near the reaction center may influence electron transfer rates, however we cannot assign any interpretable changes under physiological conditions at the current resolution.

### Cofactor environment

**Figure 4:**
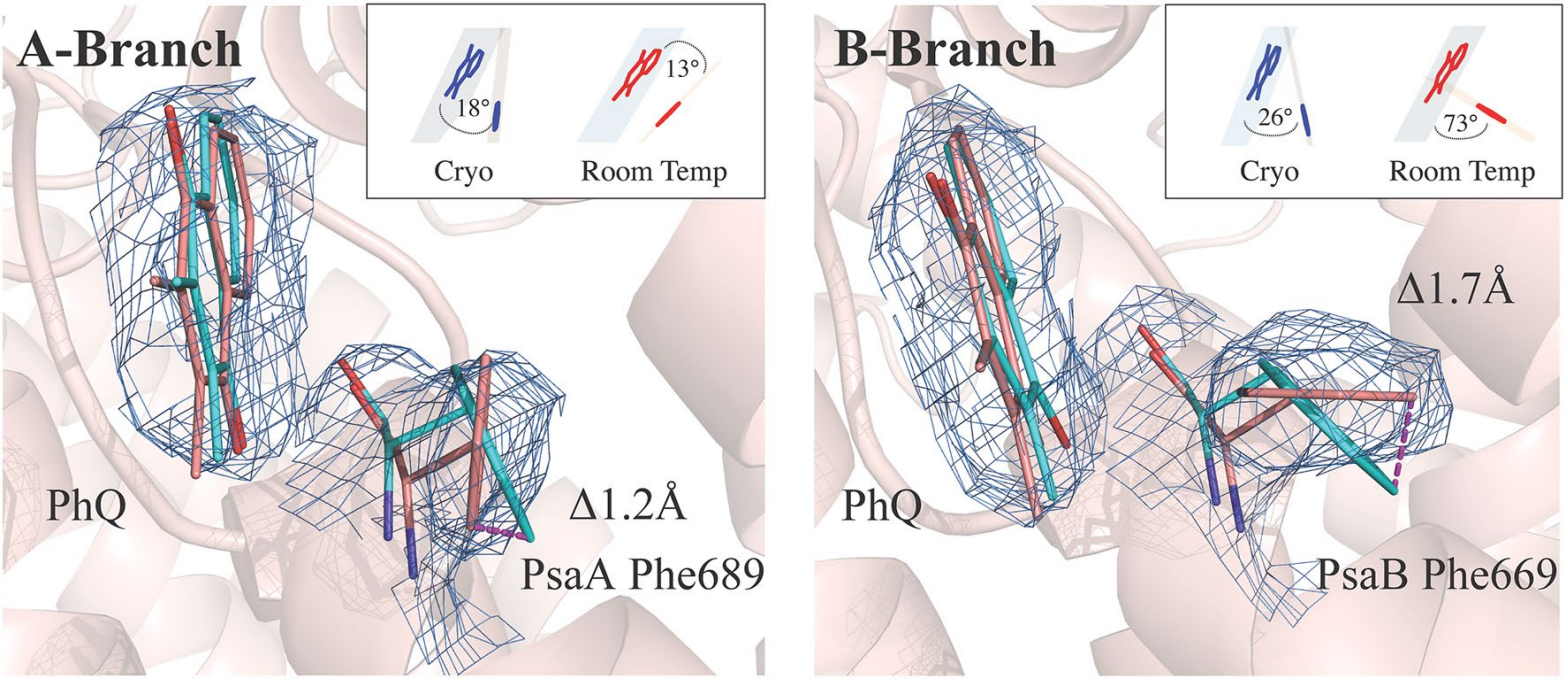
2F_obs _− F_calc_ electron density contoured to 1.5 $\sigma$ with the modeled phylloquinone in the PsaA and PsaB electron transport pathways. For aromatic residues PsaA-Trp697 and PsaA-Phe689 of PsaA and PsaB-Trp677 and PsaB-Phe669 of PsaB (salmon) the orientation obtained from the cryogenic structure (1JB0) is overlaid in cyan. PsaA-Phe689 is twisted relative to the cryogenic structure to a more parallel orientation with the phylloquinone. The B site reveals PsaB-Phe669 in an orientation more perpendicular to the plane of the phylloquinone. The stick representation of the PsaB-Phe669 and PsaA-Phe689 aromatic ring plane angles to phylloquinone in room temperature structure versus 1JB0 is shown in the inset. Both sites reveal significant shifts of atomic coordinates. Figure generated with Pymol v. 2.4.0a0. https://pymol.org.

Although most binding pockets for the central cofactors of the electron transfer chain were found to be unchanged by the difference in temperature, upon careful comparison between the cryogenic structure (1JB0)^1^ and the highest quality 2.7 Å resolution XFEL structure collected at RT, some subtle but distinct differences were noticed. The most prominent differences were seen in the environment of the phylloquinones in both the A and the B branch, that may have implications for the directionality of electron transfer. Residues PsaA-Phe689 and PsaB-Phe669, which are immediately next to the phylloquinones PhQ_A_ and PhQ_B_ respectively, shift in position along with a twist in the orientation of the phylloquinones, with some atomic positions changing greater than 1.0 Å (Fig. 4).

In order to exclude modeling errors several controls were performed. These include omit map generation (Fig. S6), repositioning PsaB-Phe669 in the cryogenic position and running systematic refinements of these phenylalanine residues in multiple rotated conformations in Phenix (Fig. S7), and F_obs_ − F_calc_ map inspection. All of these controls indicated that side chain position of PsaB-Phe669 in the 2.75 Å resolution room temperature structure is different from the positions modeled in the cryogenic data.

The angle between the planes of the ring structures of the phenylalanines and the plane of the phylloquinones ring structures changes in the A-branch by 5° and becomes more parallel (13 ± 8° at RT), increasing the off-center π-stacking interaction and the distance to 3.9 Å. In contrast, PsaB-Phe669 rotates out of plane by 47° and becomes more perpendicular in the B branch (73 ± 6° at RT) with the distance reduced to 3.3 Å. The positional precision of these assignments was estimated by perturbation of the structure factors using END/RAPID^39^. This method allows us to assign coordinate error to individual atoms. In short, we randomly perturbed the structure factors by a factor in the range of ± [F_obs _− F_model_] in 100 trials, and re-refinement was carried out for each perturbed dataset. The standard deviation of aromatic plane angles was obtained from this set of 100 structures, confirming our reported positions (Fig. S8). The two tryptophans PsaA-Trp697 and PsaB-Trp677 similarly pi-stacked to the phylloquinones are unchanged, with a distance of 3 Å and angles of 16° and 13° to the phylloquinones, respectively. Both the tryptophans and the phenylalanines are part of a network of aromatic side chains within π-interacting distance of 3.5–4.5 Å that spans from the antenna chlorophyll (1230, B-branch numbering) and its histidine ligand (PsaB-His718) via the Phe, PhQ Trp, and A_0_ to PsaB-Tyr676. Typical phenyl–phenyl interaction energies were reported to be in a similar range as H-bonding^40^.

**Figure 5:**
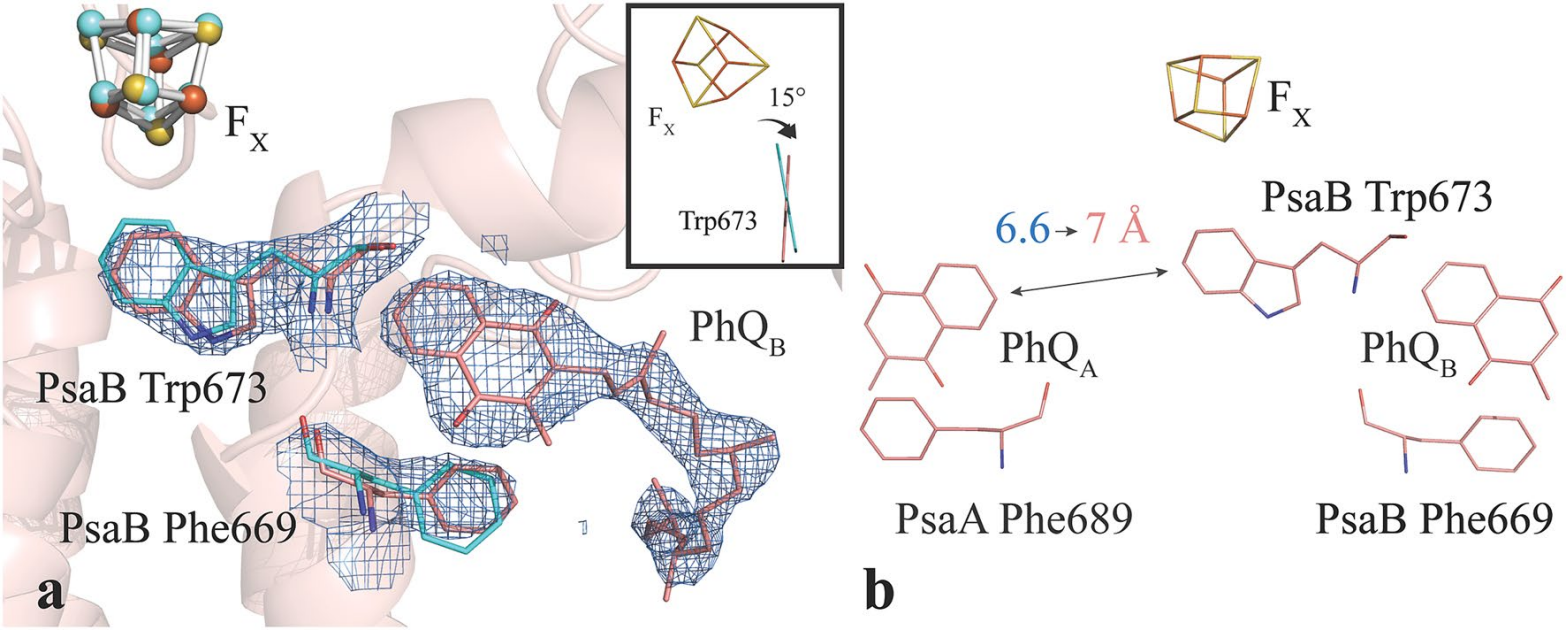
The upper part of the electron transfer chain in PS I. (** a**) Upper B branch of the electron transfer pathway modeled into 2F_obs _− F_calc_ electron density contoured to 2 $\sigma$. Also shown are the aromatic amino acids PsaB-Trp673 and PsaB-Phe669 in proximity to the phylloquinone. The plane of Trp673 has twisted 15° relative to the cryogenic structure (inset). (**b**) Stick representation of the upper A and B branches of the reaction center including the [4Fe4S] cluster F_X_ and the phylloquinones PhQ_A_ and PhQ_B_. The distance of PhQ_A_ to the symmetry breaking PsaB-Trp673 is with 7 Å in the room temperature structure longer than the 6.6 Å in the cryo structure. The distance between F_X_ and the quinones is not altered. Figure generated with Pymol v. 2.4.0a0. https://pymol.org.

Another difference in the cofactor environment is observed in the B branch between PhQ_B_ and F_X_. The conformation of PsaB-Trp673 is twisted by 15° (Fig. 5a). In this orientation, the angle between the ring planes of Trp and PhQ_B_ is 50 ± 2° in the room temperature structure. Also, the distance of the PsaB-Trp673 to PhQ_A_ at 7 Å is longer in the room temperature structure (Fig. 5b, 6.6 Å in the cryo structure). Interestingly, this does not alter the distance of either quinone to F_X_.

Examination of the recent 2.9 Å structure of *T. elongatus* PS I collected at the European XFEL^29^ did not reveal the same structural changes we observed. This main aim of this study seems to be to demonstrate the feasibility of membrane bound protein data collection at the European XFEL and not much structural refinement of the PS I structure was described in the publication.

In order to lay the groundwork for future illumination driven experiments and probe for structural intermediates in the light-induced electron transfer chain of PS I, we devised a method to measure a PS I structure under conditions that will ensure fast re-reduction of P700 upon illumination. To this end, we treated PS I crystals with ascorbic acid (see Methods) and performed the RT XFEL measurements under dark conditions using the DOT setup installed at the NCI instrument of the PAL-XFEL^41, 42^. Diffraction data to 2.8 Å resolution were obtained and merged to a resolution of 3.0 Å (“dark/reduced’, see Table 1). This structure exhibited the same general expansion features as the 2.75 Å dark state structure without ascorbic acid present (Fig. S4) and no specific differences due to the ascorbate treatment were found.

## Discussion

With the capabilities of XFELs, one can now conduct X-Ray Diffraction (XRD) studies under physiological conditions^43, 44^. In addition, the time structure of the XFELs allows one to perform time-resolved studies in time scales that are not accessible with other sources^45^. Recent time-resolved work at LCLS has included the study of many dynamic processes in proteins occur in the sub-ps and ps time scales^46–48^. A series of recent ~ 2.9 Å resolution SFX structures of a photosynthetic reaction center from *B.virdis* has revealed conformational response to various ultrafast time points, with positional shifts of up to 0.3 Å reported at the special pair^49^.

Room temperature XFEL studies have also reported differences in structures of protein complexes compared to data collected at cryogenic temperatures. Young et al. showed at ~ 3 Å resolution, that the dimeric complex in photosystem II is slightly extended (~ 0.5 Å) at room temperature compared to structures at cryogenic temperatures^32^. This expansion was pronounced along the membrane plane and less so perpendicular to the plane. We observe a similar trend in our room temperature PS I data from data collected at four different beamlines (LCLS, SwissFEL, SACLA, and PAL), on PS I crystals from different preparations, with a clear expansion along the membrane plane (Fig. 3 and S4) and only minimal changes perpendicular to the membrane.

One possible explanation discussed for this anisotropic expansion in our earlier study on PS II was that the lipid molecules that are bound inside the dimeric protein complex show higher mobility at room temperature compared to cryogenic temperatures^32^. In comparison to PS II the number of lipids located inside the studied PS I complex is smaller (four instead of 25 lipid molecules per monomer) (Fig. S9a). But in addition to the hydrophobic fatty acid tails, other aliphatic chains of molecules bound inside the protein complex should be taken into account. Counting the isoprenoid chains of Chls, pheophytins and quinones, as well as the hydrophobic chains of detergent molecules (which most likely replace native lipids) in addition to the fatty acid chains of lipids, an average of 3–4 chains per transmembrane helix (TMH) is found for both PS I and PS II (Fig. S9b).

When assuming the tilt of the TMH with respect to the membrane normal is not changing with temperature, the dominant factor determining the expansion of the PS I protein complex along the membrane normal is the thermal expansion of the hydrogen bonding interactions within each transmembrane alpha helix, and a thermal expansion coefficient of 2 * 10^–4^ K^−1^ was observed for these bonds^50^. For lipid bilayers, an increase of the area per molecule with increased temperature is commonly observed and this is attributed to the higher mobility of the fatty acid chains at higher temperature (see e.g.^51–53^). The thermal expansion coefficient of lipids in the lateral direction of the lipid bilayer was found to be about 2 * 10^–2^ K^−1^^53^, substantially larger than the thermal expansion observed for H-bonding interactions in α-helices. Assuming that the aliphatic chains behave similarly, we therefore suggest that the thermal mobility of the 3–4 aliphatic units present for each TMH are responsible for the observed preferential thermal expansion of the complex in the membrane plane direction.

Although the resulting changes in Chl distances in PS I at room temperature versus the cryogenic structure are subtle, they should be considered when calculating exciton transfer rates within the antenna system and between the antenna and the RC of PS I at room temperature, because small changes in distances can have a large effect on the electron transfer rates. Importantly, the position of the connecting Chls between the internal antenna system and the reaction center were found to be unaffected by the change in temperature, suggesting that the overall coupling for excitation energy transfer is not strongly influenced by temperature. We also did not observe any changes in the proposed clusters of red-shifted Chls in PS I at room temperature (Fig. S10), indicating that the conclusions regarding the position of long-wavelength absorbing Chl in PS I^54, 55^ drawn based on the cryogenic structure are still valid at room temperature.

When inspecting individual binding pockets of the electron transfer cofactors in the A and B chains, no structural change around the six central chlorophyll molecules is observed between the cryogenic and the room temperature dark structures. This agrees well with a recent MD simulation predicting a very rigid local protein environment^56^. A major difference was found around the phylloquinones, which are within π-stacking distance to conserved Trp and Phe side chains in the A and B branches. The Trp side chains are offset-stacked with a distance of 3 Å and an angle of 16 and 13° relative to the quinone planes. When these Trp were mutated to Phe, the kinetics of A_1_^—^ oxidation slowed down by a factor of 3–5 at the modified branch^21^. This was attributed to a modified π-stacking and a possible change of the local environment, such as the exposure to solvent. In purely electrostatic calculations the influence of π-stacking on the redox potential of the quinones was small (30 mV)^57^. In contrast, in a quantum chemical simulation of only the quinone head group and an indole, a larger shift of the quinone redox potential was found (50–150 mV)^58^. This shift may be overestimated without the dielectric screening effect of the protein environment^57^. In the quantum chemical study, it was also found that the electro-positive periphery of the tryptophan will try to reorient towards the quinone in its reduced state. If this is prevented by the protein environment, the parallel orientation will destabilize the semiquinone anion.

Here, we report a rotation in the aromatic rings of the phenylalanines PsaA-Phe689 and PsaB-Phe669 (Fig. 4). At a distance of 3.9 Å and 3.3 Å, the rings are within the typical range of $\pi$ interactions. Such a non-parallel orientation is a common observation rather than an exception^40^. A reorientation of the ring to 73 ± 6° in the B branch exposes the electropositive ring to the quinone ring and likely contributes to stabilizing the semiquinone anion state. Since the aromatic structure of phenylalanine is smaller and the interaction distance to phylloquinone larger than for the tryptophan, however, this semiquinone stabilization effect, and in turn the effect on the redox potential will be relatively small.

Given the expected magnitude and direction of the changes, the likely role of phenylalanine orientation to the redox properties is to compensate for the larger shift induced by more drastic structural differences between two branches. These larger structural changes include differences in the backbone twist, protonation state of residues, and residue identity itself, and are predicted to be responsible for ~ 100 mV difference in the PhQ_A_ and PhQ_B_ redox potentials^59^. As a result of these structural changes, the redox potentials of PhQ_A_ is positively shifted and is thermodynamically uphill to F_X_, while PhQ_B_ is negatively shifted and is thermodynamically downhill or isoenergetic to F_X_. Such shifts in redox potentials need to be regulated, however, such that forward electron transfer from A_1_ to F_X_ is still favorable in both branches over the back reaction processes. Further increase in the PhQ_A_ potential induces P700^+^PhQ_A_^–^ recombination reaction^60^ and decreasing PhQ_B_ potential allows for a competition between A_0_ to A_1_ electron transfer and back reaction from A_0_^61^. The orientation of phenylalanine at room temperature, which would offset the potentials to more negative/positive directions for PhQ_A_/PhQ_B_, respectively, may function as a mechanism to fine tune the redox properties to prevent such over-shifting. The observation presented here suggests that the two branches may be applying different redox tuning to A_1_ by using a conserved residue with similar backbone orientation.

In a FTIR study, it is also suggested that the redox potential difference between the quinones may stem from a stronger H-bonding of PhQ_A_^—^ vs PhQ_B_^—^^62^. The above discussed rotation of PsaB-Phe669 to the reduced state stabilizing T-stacking, may weaken the PhQ_B_ H-bond at the opposite side. This is opposed to the parallel π-stacking at the A side where no counterforce to the H-bond will be developed, in line with the FTIR study. Albeit they are strictly conserved (Table S2), the phenylalanines have not been subject to many studies. The point mutation PsaA-Phe689Asn resulted in a ~ 100-fold decrease in the observed rate of PhQ_A_^—^ oxidation^63^. This extreme slowdown is unlikely to be caused by just a modification of the π-stacking. Instead, it highlights the structural importance of the two phenylalanine aromatic rings, their positioning close to the carbonyl oxygens of the quinones (shielding it from H-bonding with solvent), the binding affinity of phylloquinones and possibly their network of π-interactions including the antenna chlorophylls and A_0_.

As both PsaA-Phe689 and PsaB-Phe669 assume a more parallel orientation with PhQ in the cryogenic structure, the effect of redox tuning and on H-bonding strength for PhQ_B_ is likely diminished at lower temperatures. At cryogenic temperature, electron transfer in PS I becomes highly heterogeneous: a significant fraction of centers is only capable of undertaking incomplete electron transfer to PhQ or F_X_, and the fractions that achieves electron transfer to terminal acceptors are unable to recombine to the neutral ground state^64^. The heterogeneous and incomplete electron transfer processes indicate that cofactors and the surrounding environment are not necessarily frozen into the lowest energy configuration, but into multiple local minima that are unfavorable for performing efficient electron transfer (see discussions in^65^). Given the nature of PS I at different temperatures, the reoriented phenylalanine at cryogenic temperature does not imply that multiple conformers exist at room temperature and freezes into the low temperature structure. Rather, a likely scenario is that the reorientation is part of the structural changes that contribute to heterogeneity and impartial electron transfer. From the kinetic measurement, the rate of electron transfer through PhQ_B_ at cryogenic temperature is reported to be relatively temperature independent, but the fractional usage of the B branch is significantly diminished^25, 66^.

In addition to the phenylalanine, PsaB-Trp673 also rotates by 15° with respect to PhQ_B_ and the distance to PhQ_A_ is increased to 7 Å without changing the quinone-F_X_ distance significantly (Fig. 5). For the angle between Trp673 and PhQ_B_, an error of only 2.3° was found (Fig. S8). Assuming that the cryo structure is even better defined, a change of relative angle of 15° is clearly beyond this error estimate. A strong effect on the PhQ_B_^—^ reoxidation kinetics was found by time-resolved measurements when a point mutation is introduced to PsaB-Trp673^67^. This symmetry-breaking PsaB-Trp673 coordinates and separates two water clusters. Electrostatic calculations attributed the kinetic difference of the quinone oxidation to several factors associated with this residue and coordinated water molecules. The A branch counterpart to PsaB-Trp673 is PsaA-Gly693: a significant difference in the headgroup sizes induces a different twist to the backbone of these residues. This difference in twists orients a backbone carbonyl to the quinone in the B branch, and away from the quinone in the A branch. In addition, the water molecules bisected and coordinated by PsaB-Trp673 are within interacting distance with PsaB-Asp575. The carboxyl oxygens of PsaB-Asp575 are 3.3 and 2.8 Å from the closest water oxygen, and a theoretical study has predicted that the protonation state of PsaB-Asp575 influences the redox potential of PhQ_A_ by ~ 80 mV^59^. Recent FTIR studies have also shown that a mutation on PsaB-Trp673 disturbs the water cluster coordination, and results in protonation of phylloquinones^68^. However, at 2.75 Å resolution electron density may show ambiguity for water positions and highly mobile waters cannot be modelled accurately. Nonetheless, the observed increased distance between PsaB-Trp673 and PhQ_A_ will modify water positions in the cavity and the specific coordination of the water clusters on both sides of Trp673 is likely crucial to fine-tuning the redox potentials of the phylloquinones. Indeed, we find that the two water molecules that were involved in H-bonding interaction to the π-electrons of the tryptophan ring in the cryogenic structure are conserved in our RT structure and one of them is involved in the H-bonding network to PsaB-Asp575.

A comparison of structures from higher plant species such as *Pisum sativum* (pea)^4^, and cyanobacterial species such as *Synechocystis sp* PCC6803^69^, and *T. elongatus*^1, 38^*,* all collected under cryogenic conditions, reveal that the positions of PsaA- Phe689, PsaB-Phe669 and PsaB-Trp673 are relatively conserved and slightly different compared to the positions observed in the room temperature structure (Fig. S11). At room temperature, the amino acid side chains might have more flexibility to adapt to a more favorable conformation to facilitate electron transfer across the membrane plane. Such conformational sampling is likely not possible at cryogenic temperatures; thus, the subtle but clear differences observed in proximity to the central cofactor network demonstrate the importance of room temperature measurements to provide a more physiologically relevant view of the reaction center environment.

Additionally, conditions established here describe data collection in the presence of ascorbic acid, which will aid in experiments to study the characteristics of specific reduced or oxidized electron transfer cofactors. Under these conditions the same structural changes at room temperature are found and confirm the above presented observations.

To summarize, the radiation damage-free room-temperature structure of PS I reveals that the overall membrane protein complex is slightly expanded along the membrane plane, compared to previous cryogenic measurements, and that specific differences of the aromatic side chains PsaA-Phe689, PsaB-Phe669, and PsaB-Trp673 exist in the binding pockets of the two phylloquinone electron acceptors in both branches. These structural differences can have direct consequences on the redox properties of these cofactors and could be partly responsible for the observed differences in electron transfer rates along the two branches. A combination of improvements in crystallization and data collection methods has allowed improving the resolution for time-resolved RT measurements of PS II from ~ 6 Å to close to 2.0 Å. This development, together with the already obtained initial data quality that has already been obtained for PS I presented here, is encouraging for future time-resolved studies that will allow us to elucidate the nature of protein-mediated electron transfer in PS I.

## Methods

### PS I preparation and crystallization

PS I was prepared as described previously^70^. BN-PAGE and MALDI-TOF revealed that PS I was in its trimeric state and no subunits were lost during the purification. Dynamic light scattering revealed a hydrodynamic radius of 9.4 nm and a polydisperity index of 5%. Crystal growth was analyzed in a range from 7 to 18 mM Na_2_SO_4_ (Fig. S12). To ensure high quality of the protein, purified PS I was pre-crystallized in 12 mM Na_2_SO_4_, 5 mM MES pH 6.0 and 0.02% dodecyl-*β*-D-maltoside (DDM), washed and stored in the form of a suspension of microcrystals at 4 °C. On the day of the diffraction measurement the microcrystals were dissolved in the same buffer containing 40 mM Na_2_SO_4_ and a final crystallization was performed by diluting the protein to a Chl concentration of 1 mM in 12 mM Na_2_SO_4_, 5 mM MES pH 6.0 and 0.02% DDM at room temperature. The crystals grew to a size of 15–25 µm within 15–20 min (Fig. S2). Crystal growth was stopped by tenfold dilution with a salt-free buffer (5 mM MES pH 6, 0.02% DDM). For the measurement at LCLS and SACLA, the crystals were directly used in the sample delivery system. For the measurement at PAL, the crystals were treated with 5 mM ascorbic acid and stored in the dark prior to data collection.

### Sample delivery and data collection

SFX data were collected either at the MFX instrument^36^ of the LCLS facility (SLAC National Accelerator Lab, Menlo Park, CA), at the BERNINA instrument^71^ of the ARAMIS beamline at the SwissFEL FEL (Paul Scherrer Institut, Villigen, Switzerland)^35^, or at BL-2 EH3 of the SACLA XFEL facility at Hyogo, Japan^72^. In addition, data from crystals treated with ascorbic acid were measured at the NCI beamline of the PAL-XFEL facility in Pohang, Korea^41^. In all cases the suspension of microcrystals was kept in the dark while being delivered to a focusing acoustic transducer to form droplets of 4 nl as previously described^33^. The droplets were deposited on a kapton tape running at a speed of 300 mm/sec and traveled through a helium enclosure for 0.8 s before being probed by the X-ray pulse. The X-ray beam parameters for all three facilities are given in Table S1. For MFX diffraction data were detected at a rate of 10 Hz on a Rayonix MX 170HS detector with 2 × 2 binning at a distance of 205 mm from the interaction point. At the SwissFEL a 16 Mpixel Jungfrau detector placed at a distance of 163 mm was read out at 25 Hz. At SACLA diffraction data were recorded on an MPCCD octal detector at a rate of 30 Hz and at PAL on a Rayonix MX 225HS with a 3 × 3 binning at 145 mm distance at 15 Hz.

### Data processing, model building and map calculation

Data were processed using *cctbx.xfel* and DIALS^73–75^. *cctbx.xfel*^74, 76, 77^ was custom built for NERSC using instructions here: https://gitlab.com/NERSC/lcls-software/-/tree/beamtime-2020-09/cctbx-production. Note that the branch referenced is specific to this beamtime. The data was batched by timestamp, and joint refinement of the crystal models against the detector position for each batch was performed to account for small time-dependent variations in detector position^74^. Integrated intensities were corrected for the kapton tape shadow as described in Fuller et al.^33^. Data from LCLS and SwissFEL were co-scaled and merged using *cctbx.xfel.merge* with errors determined by the method of Evans as described by Brewster et al.^78, 79^. https://gitlab.com/NERSC/lcls-software/-/tree/beamtime-2020-09/cctbx-production.

Indexing solutions for serial crystallographic data in the merohedral space group P6_3_ are inherently ambiguous; this ambiguity was resolved by an implementation in the *cctbx.xfel.merge* processing pipeline of the algorithm of Brehm and Diederichs^80^, supported by coset decomposition as described by Gildea and Winter^81^. The full Gildea-Winter algorithm has N^2^M^2^ complexity; i.e. correlations are computed between every pair of shots after transforming by every candidate reindexing operator. For 100,000 shots in P6_3_, this requires computing ~ 4 × 10^10^ correlation coefficients, which is an intractable problem with current technology. We reduced the algorithmic complexity to NM^2^ by splitting the data into tranches of 200–300 shots for clustering analysis; these tranches were distributed across MPI ranks for multiprocessing; and were constructed with overlapping subsets of shots, so that tranch results could be reconciled at the conclusion. When the computation is performed on 400 tranches of 250 shots, a total of ~ 10^8^ correlation coefficients are needed, a 400 × reduction in the problem size. Merging was performed on several hundred MPI ranks and the total time was typically < 10 min per data set. A full usage guide for this method (implemented as a *cctbx.xfel.merge* worker) is available. https://github.com/cctbx/cctbx_project/blob/master/xfel/merging/application/modify/README.md.

A multistep approach was used to determine resolution cutoffs^82^, where for each separate image and lattice, assessment of the outer resolution bin for detectable spot intensities was conducted. Bragg spot intensities I and standard deviations σ(I) for one lattice were obtained using standard 2D summation integration. Final resolution cutoff for the merged data set was quantified using CC_1/2_^83^, with the resolution limit upper bin being defined by the point where CC_1/2_ no longer decreases monotonically, indicating no useful information is obtained beyond this point.

Molecular replacement was carried out using Phaser-MR^84^ within the Phenix package^85^ using the deposited cryogenic structure 1JB0^1^ as a reference model. Model building was performed in Coot^86^ with multiple iterations of refinement using *phenix.refine*^85, 87, 88^. Waters lacking clear density were removed from the 2.75 Å LCLS/SwissFEL structure, and all waters not coordinated to ions were removed from the lower resolution structures. Restraints were generated using elbow^89^ within the phenix package with some modifications, and custom restraints were constructed for the special pair chlorophyll designated “chlorophyll a epimer” which is labeled PA in these structures. F_obs _− F_obs_ and F_calc _− F_calc_ maps were generated using the isomorphous difference map tool *phenix.fobs_minus_fobs_map* within the Phenix package. Data statistics can be found in Table 1.

### Coordinate error estimation

To estimate the positional precision of individual cofactors and side chains, we used END/RAPID to perturb the structure factors, as previously employed^39, 82^. The structure factors were adjusted by ± |Fobs − Fmodel| in 100 trials using the END/RAPID command line tools (https://bl831.als.lbl.gov/END/RAPID/end.rapid/Documentation/end.rapid.Manual.htm), adding noise proportional to the error in the model to generate 100 perturbed datasets for each illumination state. We then re-refined the models against each new dataset, kicking the initial atom positions in the model using the sites.shaketool in Phenix before refinement. The mean and standard deviation of selected bond distances and angles were calculated across the re-refined models^82^.

## Supplementary Information

Supplementary Information.

## Data Availability

Structure coordinates and structure factors for the LCLS/SwissFel, PAL, and SACLA PS I data sets have been deposited in the Protein Data Bank under accession codes 7M75, 7M76 and 7M78 respectively.
