# Supplementary material for: Room temperature XFEL crystallography reveals asymmetry in the vicinity of the two phylloquinones in photosystem I
Source: Sci Rep. 2021 Nov 8;11:21787. doi: 10.1038/s41598-021-00236-3 (PMC8575901; doi:10.1038/s41598-021-00236-3)
Supplement: Supplementary file 1 — Supplementary Information. [file 41598_2021_236_MOESM1_ESM.pdf]

Supplementary Information for

Room Temperature XFEL Crystallography reveals asymmetry in the vicinity of the two phylloquinones in Photosystem I

Keable, Kölsch, Simon et al.

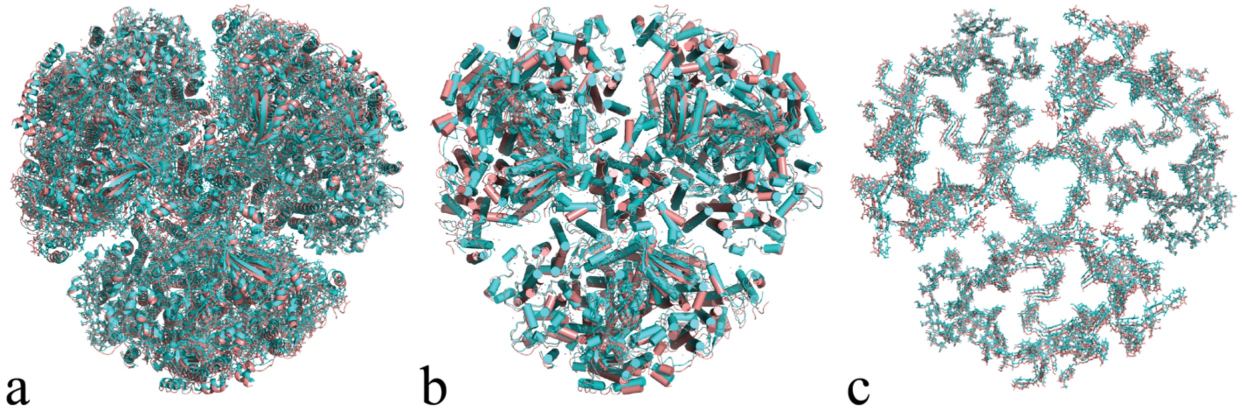

Supplementary Figure 1. Comparison between the cryogenic and room temperature structure of the PS I trimer. **a** Overlay of the cryogenic (cyan) and room temperature (salmon) structure of PS I, view is onto the membrane plane from the cytoplasmic side. **b** Same as in **a** but only protein is shown and helices are depicted as cylinders to better illustrate the shift of helices between the two structures. **c** Same as in **a** but only Chl are shown to better illustrate changes in Chl positions due to temperature change.

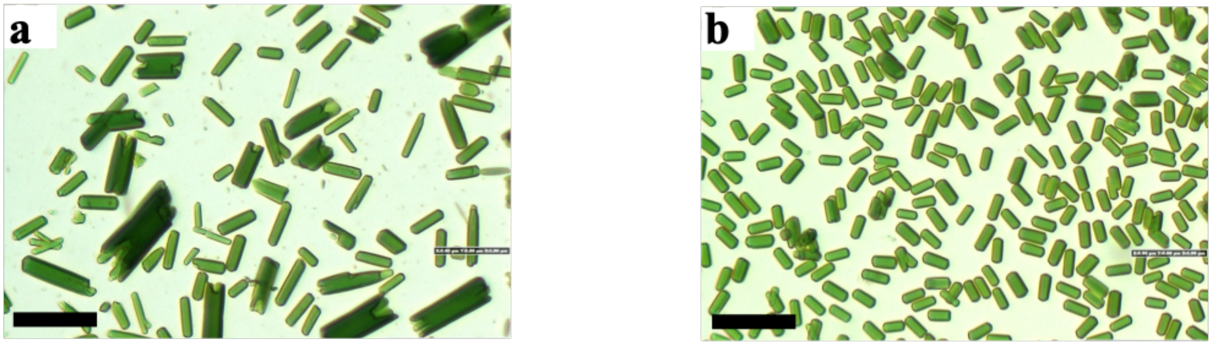

Supplementary Figure 2. Crystals of PS I. a Purified PS I protein was crystallized as per 'Materials and Methods' in reference[1]. **b** PS I micro crystals used for SFX measurements room temperature in this work. The bar represents 50 µm.

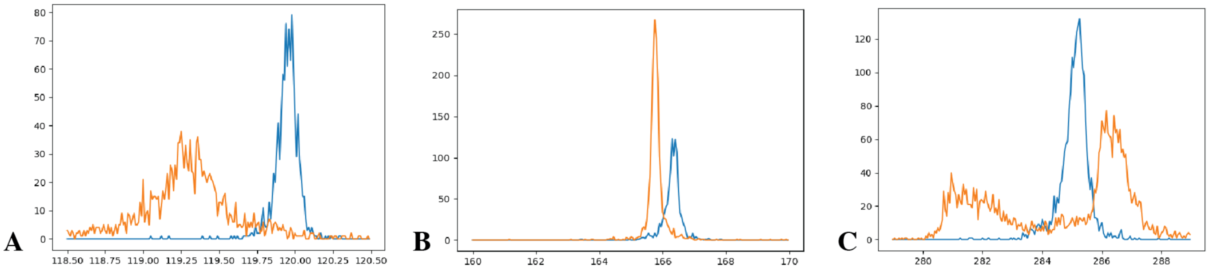

Supplementary Figure 3. Histogram of unit cell distributions. Orange, reprocessed data from CXIDB 111[2]; blue, present work. **a** β, degrees. **b** *b* axis, Å. **c** Combined histograms for *a* and *c* axes, Å. Note that these values are for the conventional setting (*b* unique) for space group $P2_1$. In all other discussions of the present hexagonal structure, we use the conventional setting for $P6_3$ with *a*=*b* and *c* unique.

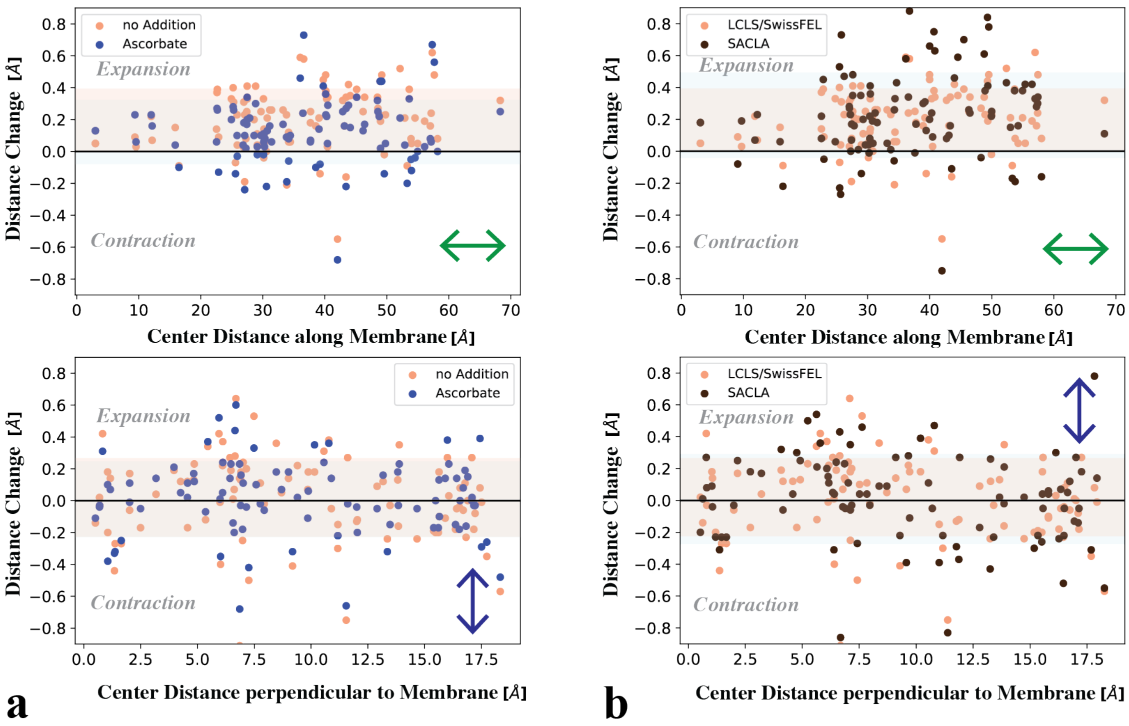

Supplementary Figure 4. Distance change (RT minus cryo) of the chlorophylls to the center between P700 and F_X. Changes are shown either along the membrane (*top*) or perpendicular to it (*bottom*). Blue markers represent the data recorded at the PAL XFEL facility of PS I with ascorbate added, salmon data without it as in fig. 3. Dark brown markers represent a control data set recorded at the SACLA XFEL facility. Positive values correspond to expansion, negative to contraction at room temperature. The 1-σ region around the average is highlighted. All data sets of the room temperature structures show an average extension by about 0.2 Å along the membrane plane. Also, the distance change perpendicular to the membrane plane is distributed symmetrically around the zero-line independent of ascorbate addition or data set.

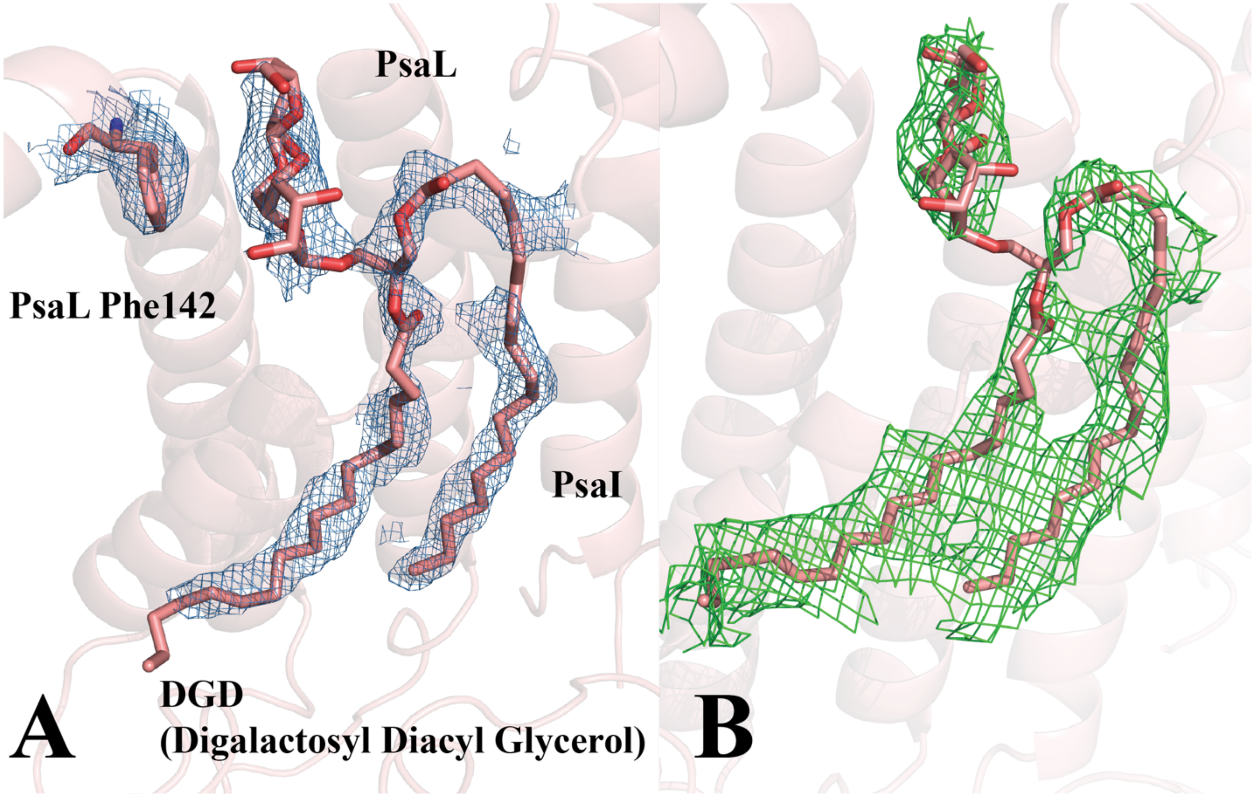

Supplementary Figure 5. Example of DGD lipid modeled into electron density. a $2F_{obs}$-F_{calc} electron density contoured to 1.0 σ with the lipid DGD modeled next to subunits L and I. Electron density is visible for the head group and the hydrophobic tails of this lipid. **b** Polder omit F_{obs}-F_{calc} electron density contoured to 2.0 σ around the modeled DGD lipid.

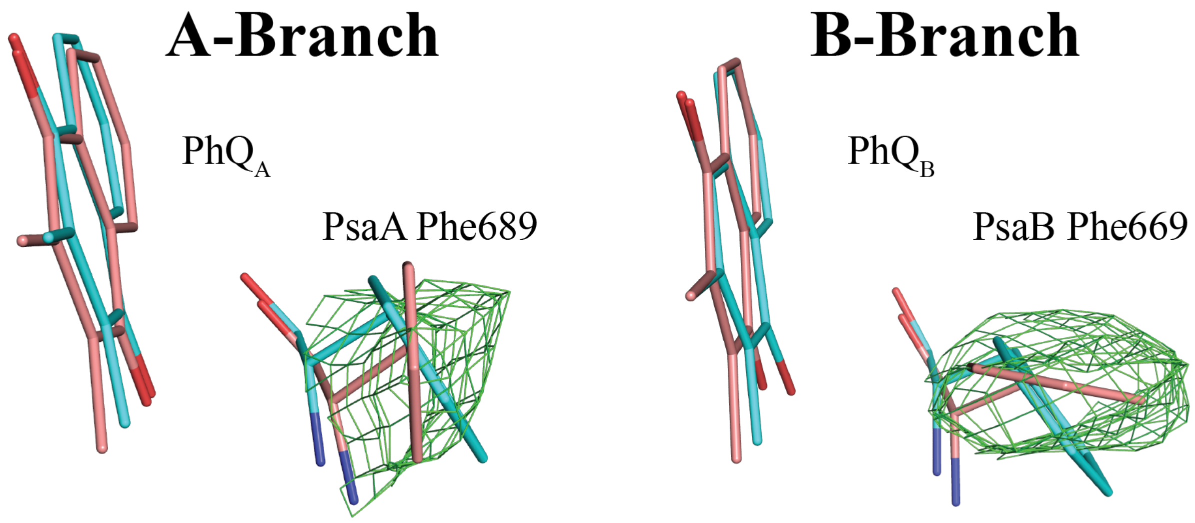

Supplementary Figure 6. Omit map of select reaction center residues. F_O-F_C omit electron density contoured to 4σ of residues Phe689 and Phe669 in the electron transfer pathway. Map generation was conducted using phenix.maps by deletion of the residues and recalculating phases and F(model).

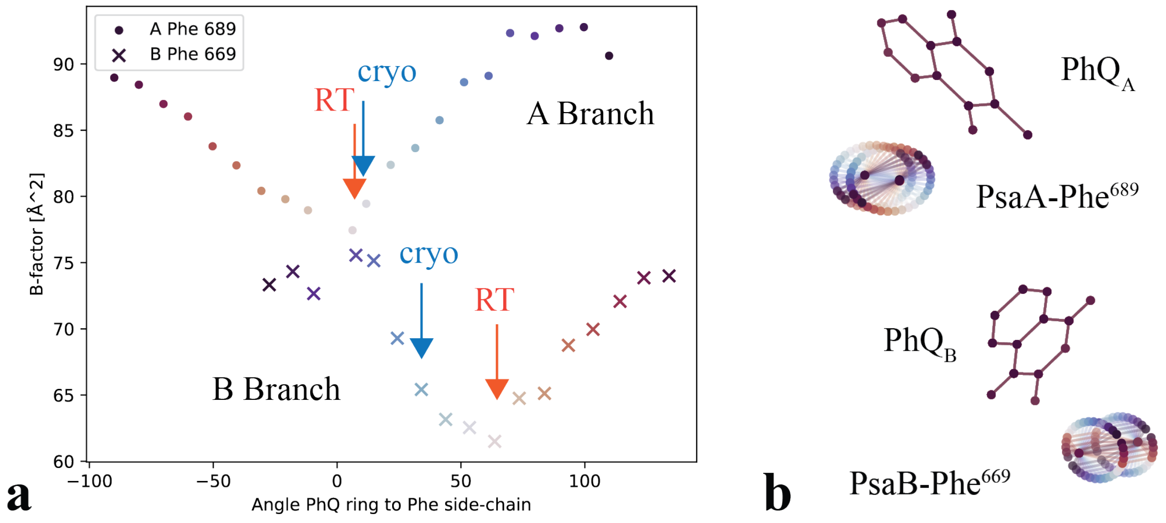

Supplementary Figure 7. Averaged B-factors of the ring carbons extracted from rotated Phe residues. To investigate whether the refined structures of the PhQ and their π-stacked PsaA-Phe689 and PsaB-Phe669 allow different rotamers or are flexible in their rotation, we refined several rotamer conformations of the phenylalanines in the room temperature structure. Only the temperature factors (B-factors) were refined to compensate for mismatching electron densities. **a** Plotted is the averaged B-factor of all ring carbon atoms of the phenylalanine versus the angle between the rings of Phe and $PhQ_{A/B}$. The starting angle to the PhQ is plotted in the lightest color. A rotation of the ring planes away from these best-refined positions (shown in **b**) results in higher B-factors which indicates the refined position in the pdb-file is the most favorable. The value of the B-factor and the width of the well on the A branch is higher and wider than on the B-branch indicating higher flexibility as well as a less defined rotation angle. We do not find indications for a second conformation on either side.

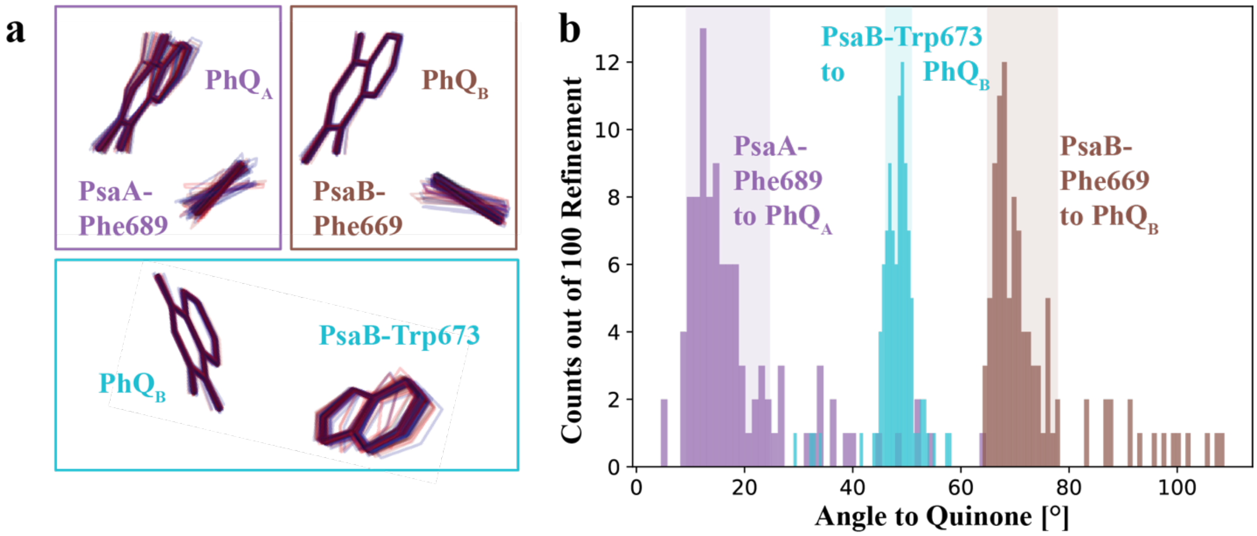

Supplementary Figure 8. Local error estimation of the relative angles of the aromatic side chains and the corresponding quinones using the END/RAPID tools. a Overlay of all 100 refinement trials from the END/RAPID protocol of the quinones and sidechains discussed in figure 4 and 5. **b** Histogram of the angles found between the aromatic sidechains and the corresponding quinones. The 1-σ region around the average is highlighted. To calculate the average and standard deviation, outliers outside a 2-σ region of the unselected data were rejected (6, 8, 6 trials rejected for angles with Phe689, Phe669, Trp673, respectively).

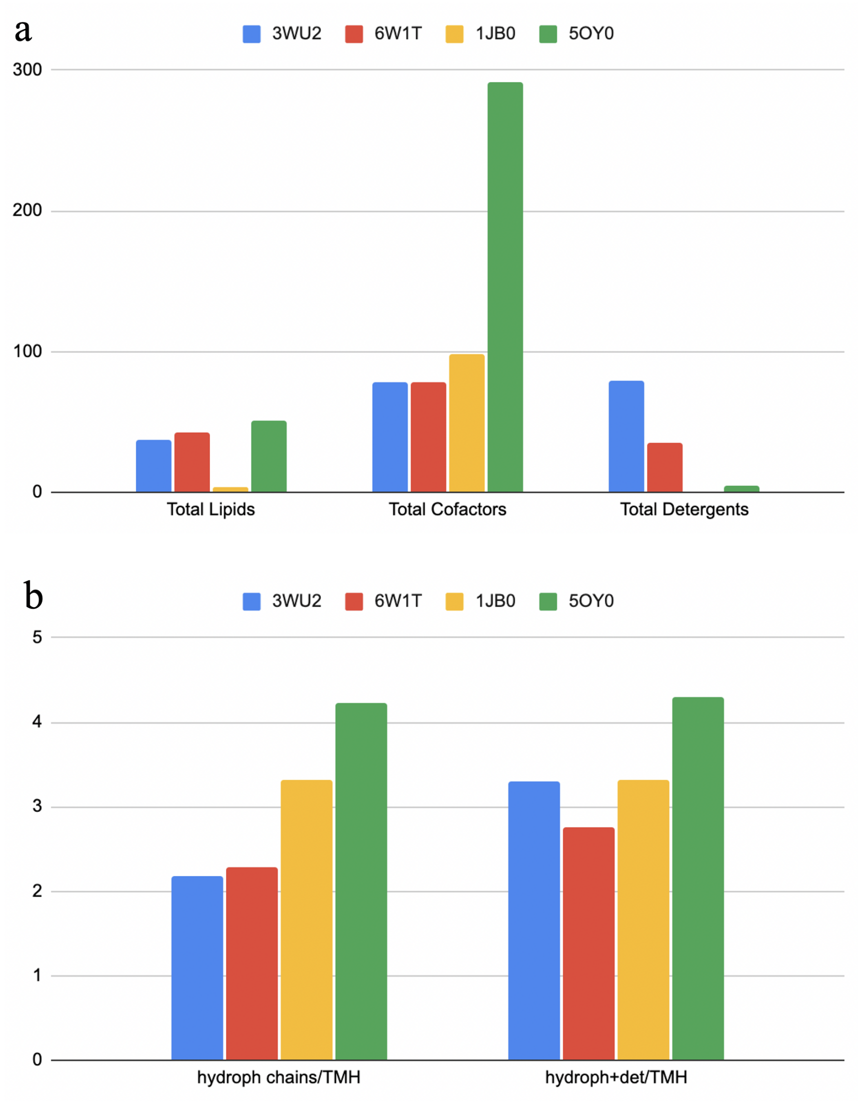

Supplementary Figure 9. Quantification of hydrophobic moieties in PSII and PSI. a The total number of lipids, cofactors that have isoprenoid chains (chlorophyls, pheophytins, plastoquinones and phylloquinones) and of detergent molecules are given for two structures of PSII from *T. elongatus* (pdb ID 3WU2, 6W1T, per dimer) as well as for the cryo structures of PSI from *T. elongatus* (pdb ID 1JB0, per monomer) and from *Synechocystis* (pdb ID 5OY0, per trimer). **b** The number of hydrophobic chains (two for each lipid, one for each detergent or cofactor) in relation to the number of transmembrane helices (TMH) present in the complex for the two PSII and the two PSI structures. On average 3-4 hydrophobic chains are found per TMH in these complexes.

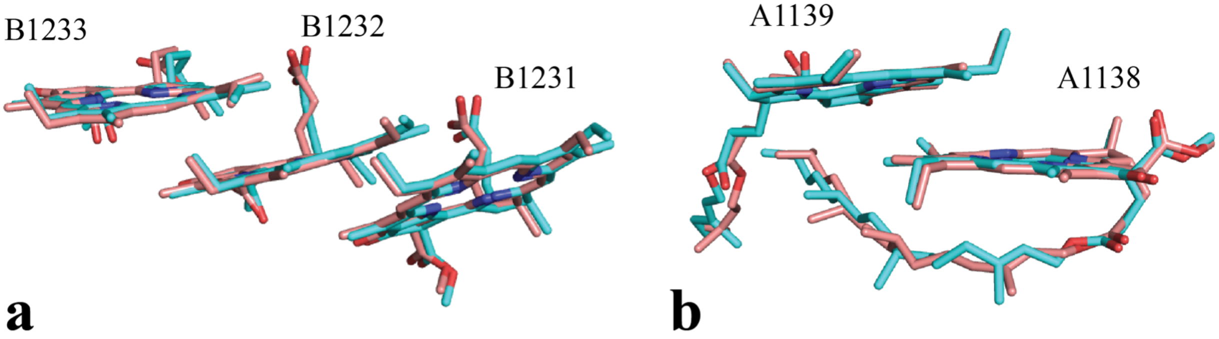

Supplementary Figure 10. Overlay of red-shifted chlorophylls. Superimposition of the cryo 1JB0 structure (cyan) and room temperature XFEL structure (salmon) showing examples of **a** a red-shifted chlorophyll trimer and **b** a red-shifted chlorophyll dimer. The two models display highly conserved positions of the red-shifted chlorophylls.

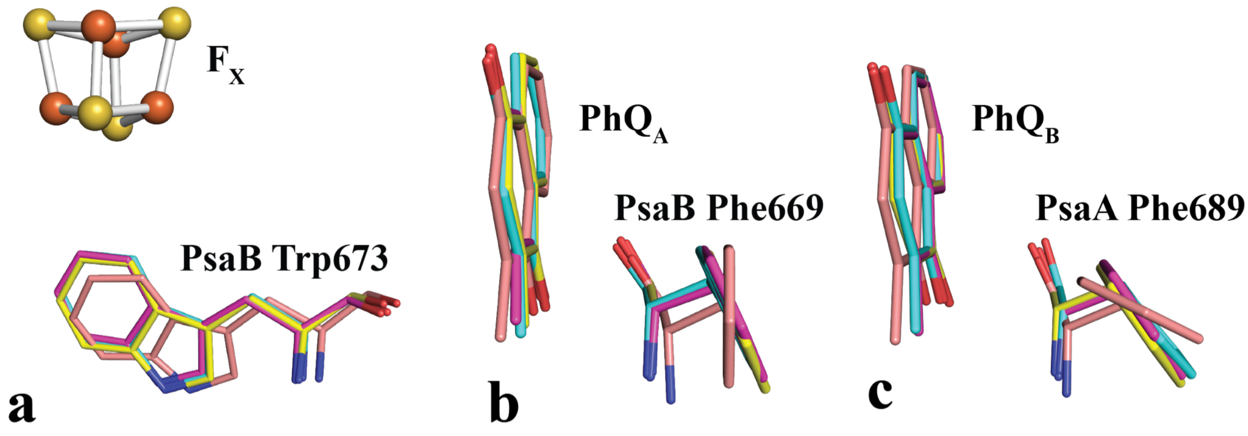

Supplementary Figure 11. Superimposition of structures from various species. Superimposition of room temperature structure (salmon) to cryogenic structures 5L8R (*Pisum sativum,*cyan), 5OY0 (*Synechocystis sp.* PCC6803, magenta) and 1JB0 (*Synechococcus elongatus*, yellow) highlighting the positions of **a** Trp673 **b** Phe669 and **c** Phe689. In contrast to the room temperature structure, all three cryogenic structures are aligned in similar orientations.

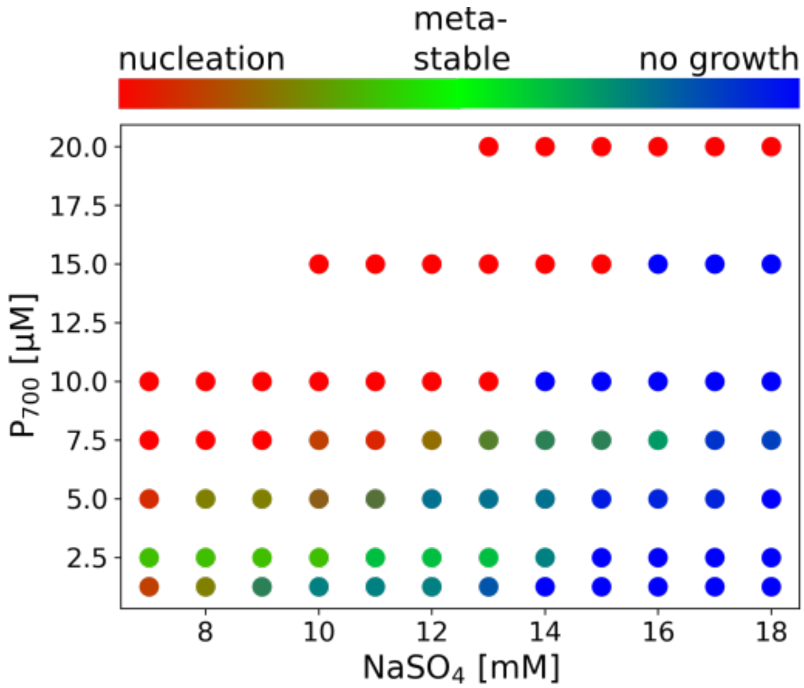

Supplementary Figure 12. Phase diagram of PS I crystallization. PS I crystals were grown in 5 mM MES buffer pH 6, 0.02 % DDM and 7 to 18 mM Na_2SO_4. Spontaneous crystal growth (red), crystal growth after addition of crystal seeds (green) and no crystal growth (blue). Mixed colors represent deviations from triplicate measurements, e.g. brown indicates that crystals grew spontaneously in two measurements but grew only after the addition of seeds in the third measurement.

Supplementary Table 1. Merging statistics for individual data sets from LCLS and SwissFEL.

Beamline	LCLS/MFX	SwissFEL/Bernina
Resolution range refined (Å)	47.76 - 2.75	56.70 - 2.75
Resolution range upper bin (Å)	(2.79 – 2.75)	(2.79 – 2.75)
Wavelength (Å)	1.306	1.306
Space group	$P6_3$	$P6_3$
Unit cell parameters (Å)	a=285.6±0.2<br>b=285.6±0.2<br>c=166.7±0.2	a=285.4±0.3<br>b=285.4±0.3<br>c=166.5±0.2
Lattices merged	51716	80243
Unique reflections	199993	195512
(upper bin)	(9928)	(7338)
Completeness	99.95%	97.99%
(upper bin)	(99.48%)	(74.08%)
$CC_{1/2}$	99.0%	97.8%
(upper bin)	(1.2%)	(4.4%)
Multiplicity	258.90	199.67
(upper bin)	(8.21)	(2.51)
$I/\sigma_{Br19}(I)$##	2.7	2.5
(upper bin)	(0.1)	(0.1)

##: as defined in [Brewster 2019][3]

1JB0| *Synechococcus elongatus*	AFSLMFLFSGRGYWQ	698
5OY0| *Synechocystis sp.*	AFSLMFLFSGRGYWQ	694
6JO6| *Chlamydomonas reinhardtii*	AFSLMFLFSGRGYWQ	694
5ZJI| *Zea mays*	AFSLMFLFSGRGYWQ	693
5L8R| *Pisum sativum*	AFSLMFLFSGRGYWQ	701
sp| *Nicotiana tabacum*	AFSLMFLFSGRGYWQ	693
sp| *Spinacia oleracea*	AFSLMFLFSGRGYWQ	693

a

1JB0| *Synechococcus elongatus*	ATGFMFLISWRGYWQ	678
5OY0| *Synechocystis sp.*	ATGFMFLISWRGYWQ	669
6JO6| *Chlamydomonas reinhardtii*	ATGFMFLISWRGYWQ	673
5ZJI| *Zea mays*	ATGFMFLISWRGYWQ	672
5L8R| *Pisum sativum*	ATGFMFLISWRGYWQ	672
sp| *Nicotiana tabacum*	ATGFMFLISWRGYWQ	672
sp| *Spinacia oleracea*	ATGFMFLISWRGYWQ	672

b

Supplementary Table 2. Sequence alignment of select reaction center residues. a Sequence alignment of PsaA showing highly conserved Phe689 (yellow) and the Trp697 (turquoise). **b** Sequence alignment of PsaB showing highly conserved residues Phe669 (yellow), Trp673 (magenta) and the Trp677 (turquoise). Sequence alignment carried out using Clustal Omega[4].

Supplementary Table 3. Experimental Parameters

		LCLS	SACLA	PAL	SwissFEL
**X-ray**	E [keV] / wavelength [nm]	9.5 / 0.131	10.5 / 0.118	9.7 / 0.127	9.5/0.131
	Pulse duration [fs]	35	10	30	50
	E_{pulse} [µJ]	4 000	310	700	200
	RepRate [Hz]	10	30	15	25
	Focus [µm]	3 x 3	2.1 x 2.2	2 x 2.3	1.2 x 1
**Detection**	Detector	Rayonix MX 170HS	MPCCD octal	Rayonix MX 225HS	JUNGFRAU 16M
	Diagonal [mm]	240	145	318	307
	Distance [mm]	205	77.5	145	166

Supplementary References

1. Kölsch A*, et al.* Insights into the binding behavior of native and non-native cytochromes to photosystem I from *Thermosynechococcus elongatus*. *J Biol Chem* **293**, 9090-9100 (2018).

2. Gisriel C*, et al.* Membrane protein megahertz crystallography at the European XFEL. *Nat Commun* **10**, 5021 (2019).

3. Brewster AS, Bhowmick A, Bolotovsky R, Mendez D, Zwart PH, Sauter NK. SAD phasing of XFEL data depends critically on the error model. *Acta Crystallogr D Struct Biol* **75**, 959-968 (2019).

4. Sievers F, Higgins DG. Clustal omega. *Curr Protoc Bioinformatics* **48**, 3 13 11-16 (2014).
